# Meeting the Challenges in Communication Change Intervention Development and Testing

**DOI:** 10.1093/geroni/igac060

**Published:** 2022-11-26

**Authors:** Mary Beth Happ

**Affiliations:** Center for Healthy Aging, Self-Management, and Complex Care, College of Nursing, The Ohio State University, Columbus, OH, USA

Communicating effectively with vulnerable, communication-impaired older adults is not an innate skill. While seemingly a “natural” and essential human interaction, communication consists of a set of learned, habituated behaviors and responses. Like any behavior change, changing caregiving communication behaviors requires intention and deliberative action.

In this issue, Coleman et al. (2022) describe developing and remotely implementing the *Changing Talk Online* (CHATO) communication intervention among nursing home staff. The focus of the Changing Talk communication training intervention is to help caregivers recognize and eliminate elderspeak, which is associated with increased resistance to care behaviors among nursing home residents with dementia. Changing Talk promotes the use of more appropriate, person-centered communication to reduce behavioral and psychological symptoms of dementia and the sequelae of those behaviors for both residents (e.g., psychotropic medication use) and staff (e.g., stress, job dissatisfaction, injury risk, inefficiency, turnover).

The original 3-hour, in-person Changing Talk (CHAT) communication training program demonstrated effectiveness in reducing elderspeak and resistance to care behaviors in three studies with staff and residents across 20 nursing homes (Williams et al., 2017). However, widespread, consistent, and sustained implementation of such training is hampered by nursing home staff turnover and multiple barriers to in-person continuing education scheduling and attendance. These same barriers impede implementation and sustainability of specialized communication training in acute care hospital settings as well (Gautier et al., 2022; Marshall & Hurtig, 2019).

The CHATO study represents an effectiveness-implementation hybrid design (Curran et al., 2012) in which the researchers pilot tested the effects of a clinical intervention on relevant outcomes (Williams et al., 2021) in a cluster randomized approach while observing and gathering information on implementation (e.g., participation in and completion of the program, passing rates, and leadership evaluation). This team approached the challenge of transitioning an effective educational behavioral practice change intervention (CHAT) to an online format with care and diligence by engaging key stakeholders, consultants, and educational development experts, and including interactive learner activities for adult learner engagement and practice applications. The second implementation challenge was remote delivery at the unit or nursing home level. Remote implementation required commitment from and sustained engagement with institutional leaders and project champions. The team provided accessible implementation supports and wisely encouraged and facilitated organizational tailoring of implementation to meet the unique needs and culture of each site.

This project is important both for the comprehensive use and examination of implementation strategies *and* for addressing a pervasive, yet mutable, adverse caregiving communication behavior. Clinical behavior change interventions are, by nature, multilevel and implementation evaluation and measurement is critical because these interventions depend on participation and full enactment by the clinical staff with the care recipient (i.e., person with dementia) to affect outcomes. The research team used the Expert Recommendation for Implementing Change collection of implementation strategies in this multilevel implementation. The study also collected data on implementation strategies adopted by each nursing home site to identify optimal intervention strategies and to understand variations in implementation outcomes across sites. It is an excellent and honest exemplar of intervention development, evolution, and testing.

Detailed information about program planning and implementation metrics establishes confidence in the integrity, feasibility, and usability of the online version of the CHAT program. Moreover, detailed implementation metrics provide a measure of intervention dose that can be weighted in the analysis of intervention outcomes. The data also point to areas for program improvement and reach. Staff who identified as Hispanic/Latino participated at lower rates, and there was extremely low representation of Black/African American nursing home staff. As the work moves to the inclusion of nursing homes with more diverse staff, it will be important to understand cultural aspects of elderspeak and to engage culture brokers and stakeholders in program acceptability and implementation planning. The barriers to computer literacy and access also require creative problem-solving for equitable delivery and uptake among dementia caregivers in all communities. Importantly, the knowledge generated from this pilot implementation study supports the scalability of multisite remote implementation for a large clinical effectiveness-implementation trial with representation from a more diverse sample of staff and dementia care settings.

Finally, the CHATO study highlights the importance of interpersonal communication as a target for scientific examination and intervention in dementia caregiving. In a study using smart health technology, Rose et al. (2021) focus on acoustic monitoring and mood state recognition toward de-escalating or preventing stressful encounters between the family caregiver and person with dementia that can precipitate behavioral and psychological symptoms in the person with dementia. The importance of verbal and nonverbal communication style and message content as a nonpharmacologic strategy to reduce behavioral and psychological symptoms of dementia holds potential for policy and practice change. Systematic study of the caregiver–care recipient communication patterns and interactions, and the characteristics of triggering versus positive communication approaches, will foster a standard of evidence-based, intentional, and self-aware, person-centered communication in dementia caregiving.
